# The Mixture to Be Taken as before

**Published:** 1897-01-23

**Authors:** 


### Editor's Letter-Box.

### "THE MIXTURE TO BE TAKEN AS BEFORE."

A correspondent writes: Could anything be done to induce doctors who dispense their own medicines to give up their custom of using the label for their medicine bottles bearing the ambiguous direction, "The mixture to be taken as before." I for one would be glad if they would, as I have on more than one occasion been much inconvenienced by it, and have hinted it to several of these gentlemen, but they go serenely on without taking much notice. Perhaps if someone with time and talent at disposal would write a little article on the subject it might receive the attention that I feel it deserves.

